# Synergistic Effects of Milk-Derived Exosomes and Galactose on *α*-Synuclein Pathology in Parkinson’s Disease and Type 2 Diabetes Mellitus

**DOI:** 10.3390/ijms22031059

**Published:** 2021-01-21

**Authors:** Bodo C. Melnik

**Affiliations:** Department of Dermatology, Environmental Medicine and Health Theory, University of Osnabrück, D-49076 Osnabrück, Germany; melnik@t-online.de; Tel.: +49-5241-988060

**Keywords:** autophagy, DNA methyltransferase 1, diabetes mellitus, galactose, milk exosome, milk microRNAs, oxidative stress, Parkinson’s disease, *α*-synuclein, vagal nerve

## Abstract

Epidemiological studies associate milk consumption with an increased risk of Parkinson’s disease (PD) and type 2 diabetes mellitus (T2D). PD is an *α*-synucleinopathy associated with mitochondrial dysfunction, oxidative stress, deficient lysosomal clearance of *α*-synuclein (*α*-syn) and aggregation of misfolded *α*-syn. In T2D, *α*-syn promotes co-aggregation with islet amyloid polypeptide in pancreatic *β*-cells. Prion-like vagal nerve-mediated propagation of exosomal *α*-syn from the gut to the brain and pancreatic islets apparently link both pathologies. Exosomes are critical transmitters of *α*-syn from cell to cell especially under conditions of compromised autophagy. This review provides translational evidence that milk exosomes (MEX) disturb *α*-syn homeostasis. MEX are taken up by intestinal epithelial cells and accumulate in the brain after oral administration to mice. The potential uptake of MEX miRNA-148a and miRNA-21 by enteroendocrine cells in the gut, dopaminergic neurons in substantia nigra and pancreatic *β*-cells may enhance miRNA-148a/DNMT1-dependent overexpression of *α*-syn and impair miRNA-148a/PPARGC1A- and miRNA-21/LAMP2A-dependent autophagy driving both diseases. MiRNA-148a- and galactose-induced mitochondrial oxidative stress activate c-Abl-mediated aggregation of *α*-syn which is exported by exosome release. Via the vagal nerve and/or systemic exosomes, toxic *α*-syn may spread to dopaminergic neurons and pancreatic *β*-cells linking the pathogenesis of PD and T2D.

## 1. Introduction

Parkinson’s disease (PD) has become increasingly prevalent as the population ages, being the second most prevalent neurodegenerative disorder worldwide [1,2,3,4,5,6]. First described by James Parkinson in 1817 [7], this chronic neurodegenerative disease is characterized by the loss of dopaminergic neurons in the substantia nigra (SN) of the midbrain resulting in decreased levels of dopamine in the striatum and disrupted motor control. Neuronal Lewy bodies (LB) with the aggregation of *α*-synuclein (*α*-syn) are the hallmark of this disease. Many other neuronal cell populations are also affected and account for the presence of non-motor symptoms including dementia. The main pathophysiological mechanisms of PD include mitochondrial dysfunction, oxidative stress, disturbed endo-lysosomal function and abnormal aggregation of *α*-syn [8,9,10,11,12,13]. PD patients classically display rest tremor, rigidity, bradykinesia, and stooping posture. PD can also be associated with neurobehavioral disorders including depression and anxiety, cognitive impairment resulting in dementia, and autonomic dysfunction such as orthostasis, hyperhidrosis and hyperseborrhea [8,9,10,11,12]. Whereas the genetics of PD are complex with a contribution of Mendelian (e.g., *SNCA*, *LRRK2*, *PARKIN*, *PINK1*) and non-Mendelian factors (e.g., single nucleotide polymorphisms), more common environmental risk factors including diet and alterations of the gut microbiome are in the focus of recent PD research [14,15,16,17]. Only 5–10% of PD cases are considered to involve genetic factors [8].

Since 2006, several longitudinal studies have assessed environmental or behavioral factors that seem to modify the risk of developing PD. Increased risk of PD has been associated with exposure to pesticides and consumption of milk, whereas a reduced risk has been reported in association with smoking, caffeine consumption, higher serum urate concentrations, physical activity, and use of ibuprofen and other medications [6,17,18,19].

In 2003, Braak et al. [20] recognized the involvement of both the enteric nervous system and the dorsal motor nucleus of the vagal nerve in initial PD. This observation, combined with the working hypothesis that the stereotypic topographic expansion pattern of the lesions may resemble that of a falling row of dominos, prompted the question of whether PD might originate outside of the central nervous system (CNS). A yet unidentified pathogen has been suspected that is capable of passing the mucosal barrier of the gastrointestinal (GI) tract and, via postganglionic enteric neurons, enters the CNS along unmyelinated preganglionic fibers generated from the visceromotor projection cells of the vagal nerve [20]. Indeed, increasing evidence supports the hypothesis that PD starts in the gut and spreads via trans-synaptic cell-to-cell transfer of pathology through the sympathetic and parasympathetic nervous systems to the SN and the CNS [21,22,23]. It has been demonstrated in a rat model that *α*-syn present in the human PD brain lysate, as well as distinct recombinant *α*-syn forms, are transported via the vagal nerve and reach the dorsal motor nucleus of the vagus in the brainstem in a time-dependent manner after their injection into the intestinal wall [24].

Recently, it has been shown that sensory endoderm-derived epithelial cells of the gut, known as enteroendocrine cells (EECs), contain *α*-syn and synapse with enteric nerves, thus providing a connection between the gut and the brain. It is possible that abnormal *α*-syn first develops in EECs and then spreads to the nervous system [22]. EECs are widely distributed throughout the GI tract and form the largest endocrine organ in the body playing a key role in the control of GI secretion and motility, the regulation of food intake, postprandial glucose levels and metabolism. Recent studies have shed light on EEC sensory transmission by showing direct connections between EECs and the nervous system via axon-like processes that form well-defined neuroepithelial circuits through which EECs can directly communicate with the neurons innervating the GI tract [25,26]. These findings highlight the key role of EECs in the complex and integrated sensory information and their impact on the gut–brain axis [27,28,29,30,31,32,33].

Ingested toxins and alterations in gut microbiota may induce *α*-syn aggregation and PD, however, it is not known how and when PD pathogenesis starts. The data for PD and Alzheimer’s disease (AD) suggest that a number of insults occurring early in life may lead or contribute to both diseases [34]. Furthermore, strong epidemiological evidence suggests a link between PD and diabetes mellitus type 2 (T2D) [35,36,37,38,39].

It is the intention of this review article to provide epidemiological and translational evidence that persistent intrinsic milk-derived signal transduction is a critical dietary factor promoting the *α*-syn-driven pathogenesis of PD and T2D.

## 2. Epidemiological Evidence

### 2.1. Milk Intake and Parkinson’s Disease

In 2002, Chen et al. [40] investigated associations between food intakes and PD risk in two large prospective cohorts, in which 210 incident PD cases in men and 184 in women were documented. A positive association was found between dairy intake and PD risk in men (relative risk RR = 1.8), but not in women (RR = 1.1). A marginal increase of risk was associated with skim or low-fat milk intake [40]. Park et al. [41] followed 7504 men for 30 years for incident PD in the Honolulu Heart Program. Men in the highest milk intake group (>473 mL/day) versus those who consumed no milk exhibited a 2.3-fold excess of PD (95% CI: 1.3–4.1). In the Finnish Mobile Clinic Health Examination Survey (FMC) conducted over a 41-year follow-up, milk consumed by women showed positive associations with PD risk [42]. The Greek cohort of the European Prospective Investigation into Cancer and Nutrition (EPIC) showed a significant correlation between milk consumption and PD (HR = 1.34; 95% CI: 1.14–1.58), whereas cheese and yogurt consumption showed no association [43]. A large meta-analysis of prospective cohort studies identified an increased risk for PD by milk consumption (RR = 1.45; 95% CI: 1.23–1.73), cheese (RR = 1.26; 95% CI: 0.99–1.60), but not yogurt (RR = 0.95; 95% CI: 0.76–1.20) [44]. The Nurses’ Health Study and the Health Professionals Follow-up Study confirmed an increased risk of PD with consumption of low-fat milk (HR = 1.39; 95% CI: 1.12–1.73) and milk of all fat levels (HR = 1.56; 95% CI: 1.30–1.88) [45]. Olsson et al. [46] recently studied the influence of milk versus fermented milk in Swedish PD patients. Compared to no or low milk intake (<40 mL/day), milk consumption of 40–159 mL/day showed an HR = 1.29 (95% CI: 1.07–1.56), 160–200 mL/day an HR = 1.19 (95% CI: 0.99–1.42), 201–400 mL/day an HR = 1.29 (95% CI: 1.08–1.53) and over 400 mL/day a HR = 1.14 (95% CI: 0.93–1.40). Fermented milk was not associated with PD risk [46]. Thus, recent epidemiological evidence points to a critical role of non-fermented versus fermented milk in the pathogenesis of PD (Table 1).

These epidemiological data imply that milk compared to other dairy products exhibits the highest risk for PD. It has been suggested that contamination of cow’s milk with environmental toxins and pesticides may explain milk’s adverse neurodegenerative effects [47,48,49,50], an opinion, which is less likely because neurotoxic chemicals should not be degraded by microbial fermentation of milk.

### 2.2. Milk Consumption and Type 2 Diabetes Mellitus

Insulin resistance has already been observed in children after high consumption of milk compared to equivalent protein intake by meat consumption [51]. The first meta-analysis, which investigated the effects of milk versus fermented milk and their relation to T2D is the European Prospective Investigation into Cancer and Nutrition (EPIC) [52]. EPIC shows an increased risk of T2D by milk consumption in five out of eight European countries including Germany [52]. The Framingham Heart Study Offspring Cohort [53] and the Physicians’ Health Study [54] confirm an association between milk consumption and prediabetes as well as T2D. The recent Dutch Lifeline Cohort Study exhibits a positive association between whole milk intake (150 g/day) and prediabetes as well as a relation between milk consumption (150 g/day), especially skim milk (150 g/day), and T2D [55]. Increased *β*-cell mTORC1 activity plays a critical role in the pathogenesis of T2D [56,57,58,59,60], which is normalized by metformin, an activator of AMP-activated protein kinase (AMPK) and inhibitor of the mechanistic target of rapamycin complex 1 (mTORC1) [61,62].

## 3. Milk: A Signaling System for Postnatal Growth and Differentiation

### 3.1. Milk Activates mTORC1 and Inhibits Autophagy

A newborn infant grows and thrives exclusively with human breastmilk (protein content 1.2 g/100 mL) and doubles birthweight after 180 days. A calf receiving cow’s milk (protein content 3.5 g/100 mL) doubles birthweight already after 40 days, pointing to a higher growth-promoting activity of bovine milk. On the cellular level, cell growth, anabolism and inhibition of autophagy are regulated by the central hub of metabolism, the kinase mTORC1 [63,64,65,66,67,68]. It has recently been appreciated that milk is not just food, but a maternal–neonatal signaling system activating mTORC1 of the milk recipient [69,70].

For mTORC1 activation, (a) growth factor signals such as insulin and insulin-like growth factor 1 (IGF-1) [71,72,73] and (b) sufficient supply of essential branched-chain amino acids (BCAAs: leucine, isoleucine, valine), arginine and methionine are required [74,75,76]. In addition, galactose (GAL) released after intestinal hydrolysis of the disaccharide lactose activates mTORC1 [77]. Milk consumption also increases insulin release of pancreatic *β*-cells and hepatic synthesis of IGF-1 [71,78,79,80]. In comparison to other protein sources, milk proteins provide the highest content of the BCAAs and high amounts of arginine and methionine. Palmitate, the major fatty acid of milk lipids, as well activates mTORC1 at the lysosomal membrane [81].

Taken together, multiple signaling pathways—activated after milk intake—promote mTORC1 activity and inhibit autophagy [69,70]. Increasing evidence substantiates that imbalances of mTORC1 and autophagy are critically involved in the pathogenesis of PD [82,83,84,85]. In contrast to milk-mediated mTORC1 activation [69,70], metformin has been identified as an inhibitor of mTORC1 [61,86,87,88]. Notably, metformin, the standard drug and AMPK activator in the treatment of T2D, has been suggested to serve as a neuroprotective agent for the prevention and treatment of PD [89,90,91,92,93]. Whereas chloroquine-mediated blockade of autophagy increases *α*-syn inclusions, AMPK agonists promote clearance of fibril-mediated *α*-syn pathology [94].

Notably, in contrast to milk intake, increased serum uric acid levels, coffee and green tea consumption and smoking reduce the risk of PD [95,96]. Recent evidence underlines that urate, caffeine, green tea polyphenols and nicotine all promote autophagy. For instance, uric acid administration modulates the levels of autophagy markers, increases autophagosome/autolysosome formation, and reduces *α*-syn accumulation in the midbrain of *SNCA*^A53T^ transgenic mice [97]. Uric acid not only acts as a potent antioxidant but induces autophagy activation via attenuation of mTORC1-dependent signaling ameliorating *α*-syn accumulation [97]. Caffeine, the major bioactive compound of coffee, and green tea polyphenols are known inhibitors of mTORC1 and activators of autophagy [98,99,100,101]. Nicotine, the major bioactive component of smoking, as well inhibits mTORC1 and activates the autophagy-lysosomal machinery [102,103]. Notably, exosome release is inhibited by sustained activation of mTORC1, leading to intracellular accumulation of CD63-positive exosome precursors, whereas inhibition of mTORC1 by rapamycin or nutrient and growth factor deprivation stimulates exosome release, which occurs concomitantly with autophagy [104]. Exciting research focuses on the crosstalk between exosome biogenesis and autophagy that plays pivotal roles in cell homeostasis, which is disturbed in neurodegenerative diseases [105,106].

### 3.2. Milk Exosomes: Epigenetic Drivers of Developmental Genes

**Figure 1:**
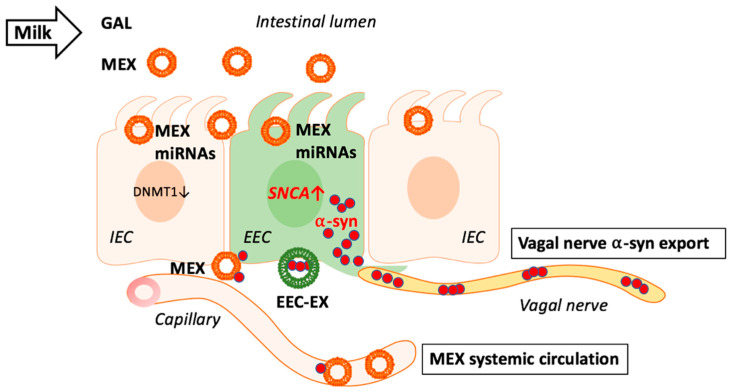
Milk exosomes (MEX) are taken up by intestinal epithelial cells (IEC) and suppress DNA methyltransferase 1 (DNMT1) resulting in promoter hypomethylation of DNMT1-methylated genes. Potential MEX uptake by adjacent enteroendocrine cells (EEC) may as well reduce DNMT1 resulting in *SNCA* promoter hypomethylation increasing the expression of *α*-synuclein (*α*-syn). Excessive or aggregated *α*-syn may leave EECs via the vagal nerve and/or MEX as well as EEC-derived exosomes (EEC-EX). MEX that reach the systemic circulation and brain may in addition transport outer exosome membrane-attached *α*-syn to target tissues.

Accumulating evidence supports the concept that milk functions as an epigenetic modifier suppressing the activity of DNA methyltransferase 1 (DNMT1) [107,108,109,110,111,112]. Fresh and pasteurized cow’s milk transfers bioactive milk exosomes (MEX) (50–100 nm in diameter) to the milk recipient [113,114,115,116,117]. MEX are highly resistant against exogenous attacks and their micro-ribonucleic acid (miRNA) content is protected by the surrounding exosomal lipid bilayer membrane. Human and bovine MEX transfer more than 400 conserved miRNAs [113,114,115,116,117]. The most abundant signature miRNAs of bovine milk are miRNA-148a and miRNA-21 [114,118]. Bovine MEX and milk extracellular vesicles (EVs) and their miRNA cargo resist digestion under simulated GI tract conditions [119]. It has been demonstrated that human and bovine MEX are taken up by intestinal epithelial cells (IECs) and vascular endothelial cells [120,121,122,123], and enhance the secretory activity of intestinal goblet cells [124]. Golan-Gerstl et al. [114] provided evidence that MEX miRNA-148a can be transferred to IECs after incubation with MEX subsequently modifying target gene expression [114] (Figure 1).

Following incubation of human MEX with normal and cancer cells, cellular expression of miRNA-148a is upregulated, whereas the expression of DNMT1 and phosphatase and tensin homog (PTEN), key target genes of miRNA-148a, are downregulated [114,125].

In addition to miRNA-148a, miRNA-21 also attenuates DNMT1 activity [126]. Notably, human and bovine miRNA-148a and human and bovine miRNA-21 have identical nucleotide sequences (mirbase.org). The maintenance DNA methyltransferase DNMT1 via CpG promoter methylation epigenetically controls the expression of important developmental genes involved in mTORC1 signaling including insulin (*INS*) [127], IGF-1 (*IGF1*) [128,129], and fat mass- and obesity-associated gene FTO (*FTO*) [130]. Of note, DNMT1 inhibition results in DNA demethylation upregulating the expression of nuclear factor erythroid 2-related factor 2 (*NRF2*) [131], a key transcription factor promoting the expression of mTOR (*MTOR*) [132].

MEX apparently orchestrate an epigenetic developmental program activating genes involved in postnatal anabolic mTORC1 signaling. After oral administration to mice, bovine MEX were detected in the intestine and accumulated in the liver and brain [133]. As MEX interact with IECs and goblet cells, it is likely that they may also be endocytosed by *α*-syn-expressing EECs involved in gut-brain communication.

### 3.3. Milk Exosomes: Potential Drivers of α-Synuclein Expression and Transmission

In early postnatal life, developmental processes are critical for establishing proper neuronal connectivity in the brain requiring synaptic machinery. One protein thought to be important in synaptic plasticity is *α*-syn [134]. Postnatal expression of *α*-syn is developmentally regulated suggesting that *α*-syn may play a pivotal role in establishing the function of basal ganglia [134]. In the rat, a high level of *α*-syn expression within cell bodies of the SN pars compacta is observed in the 1st week of postnatal life, which decreases both in intensity and number of immunoreactive cells between postnatal days 7 and 14 [134]. Soluble *α*-syn is an abundant neuronal protein that localizes predominantly to presynaptic terminals [135,136,137,138]. Monomeric *α*-syn promotes membrane curvature and assembly of the soluble N-ethylmaleimide-sensitive factor attachment protein receptor (SNARE) complex, a mediator for vesicle fusion with target membranes [139,140,141]. Of note, the SNARE protein is also the molecular basis of exocytotic activity for insulin secretion [142]. *α*-Syn contributes to synaptic tracking, vesicle budding, and vesicle recycling, while in the case of dopaminergic neurons, *α*-syn mediates dopamine synthesis, storage, and release [143,144,145]. Furthermore, SNAREs have been proposed to facilitate the fusion of multivesicular bodies with the plasma membrane promoting exosome release [105]. These observations imply that *α*-syn is of critical biological importance for postnatal growth, differentiation and function of the infant’s CNS and insulin secretion. Notably, dietary depletion of bovine MEX impaired sensorimotor gating and cognitive performance in mice [146], underlining the relationship between MEX and their functional impact on early CNS development [147].

Remarkably, exosomes have been identified as vehicles for egress of excess amounts of intracellular proteins, potentially contributing to the transfer of *α*-syn between neuronal cells [148,149,150,151]. Previous studies suggest that *α*-syn is present in exosomes from cultured cells [152]. The formation of *α*-syn oligomers and aggregates enhances the exosomal transfer of altered *α*-syn [153]. Lysosomal dysfunction increases exosome-mediated *α*-syn release and transmission [154]. *α*-Syn has been detected both within exosomes and on their outside membranes and other EVs [152], suggesting that exosomes and EVs may participate in the spreading of toxic *α*-syn species between cells. Recent evidence confirms that altered *α*-syn species shift cellular processing towards vesicular secretion [155]. EVs and exosomes apparently contribute to the spreading of harmful *α*-syn species and thereby driving the pathology of *α*-synucleinopathies [151,152,153,154,155]. Moreover, cell-derived exosomes containing *α*-syn were found to induce death of neuronal cells [156].

It has recently been hypothesized that lipid interactions play a role as trans-acting effectors in producing distinct strains of *α*-syn fibrils [157]. Of interest, exosome lipids have been shown to promote *α*-syn aggregation [158]. Aggregation of exogenous *α*-syn was accelerated by exosomes irrespective of whether they were derived from control cells or cells overexpressing *α*-syn suggesting that exosome lipids per se are responsible for this catalytic effect [149,158]. As MEX are taken up by IECs [120,121,122] and are able to cross the blood–brain barrier (BBB) accumulating in the brain [133], it is conceivable that MEX and their miRNA signaling may promote *α*-syn aggregation and spreading from the gut to the brain and other tissues.

### 3.4. Milk Exosomal miRNAs and SNCA Promoter Demethylation

Accumulating evidence underlines that hypomethylation of the *SNCA* promoter increases *α*-syn expression, which is controlled by DNMT1 [159,160,161,162,163,164,165,166,167]. Reduction of nuclear DNMT1 levels resulting in DNA hypomethylation of CpG islands upstream of *SNCA* was observed in postmortem brain samples from patients with PD or dementia with LBs [160]. The neurotoxin 1-methyl-4-phenyl-1,2,3,6-tetrahydropyridine (MPTP) is capable of producing Parkinsonism in both humans and non-human primates [168,169,170,171,172]. Notably, MPTP significantly increases the expression of miRNA-148a associated with downregulation of DNMT1 in SN of MPTP-treated mice [173]. In contrast, lithium treatment corrects the loss of nigral neurons, the increase of *α*-syn in SN as well as in the striatum of MPTP-treated mice, and decreased methylation of *SNCA* intron 1 in DNA from the same regions. In accordance, marked suppression of miRNA-148a was observed after lithium administration to MPTP-treated mice [173]. In addition, it has been shown that *α*-syn itself sequesters DNMT1 from the nucleus promoting hypomethylation of *SNCA* further augmenting *α*-syn expression in a vicious cycle [160].

Continued consumption of pasteurized milk with persistent exposure of EECs with miRNA-148a and miRNA-21 may result in overexpression of *α*-syn in EECs (Figure 1). As EVs and exosomes are able to cross the BBB [174,175,176], they may reach the brain as shown for bovine MEX orally administered to mice [133]. MEX may thus function as pathogens via modification of local (EECs) and distant (dopaminergic neurons) *SNCA* gene expression.

## 4. Autophagy-Lysosome Pathway Controls Exosomal *α*-Synuclein Export

The autophagy-lysosome pathway (ALP) regulates intracellular homeostasis of cytosolic *α*-syn and is impaired in *α*-synucleinopathies. Impaired ALP in the diseased brain not only limits intracellular degradation of misfolded proteins but also leads to detrimental microenvironmental responses due to enhanced *α*-syn secretion [177]. Whereas low-aggregated *α*-syn was predominantly released by exosomes and RAB11A-associated pathways, high-aggregated *α*-syn was secreted by membrane shedding [177]. Emerging evidence suggests that ALP influences *α*-syn via exosome/EV traffic [178]. Importantly, ALP inhibition increases the ratio of extra- to intracellular *α*-syn and upregulates *α*-syn association with EVs in neuronal cells [178]. EVs released under ALP inhibition contain higher *α*-syn cargo. Notably, EVs of cerebrospinal fluid (CSF) with characteristics of exosome markers (CD63, CD81) transfer *α*-syn from cell to cell in vivo [178]. Thus, inhibition of macroautophagy/autophagy enhances *α*-syn spreading via exosome/EV release [178].

Oxidative stress-induced mitochondrial dysfunction and neuronal cell death have important roles in the development of neurodegenerative diseases. Fibroblasts from sporadic PD patients show hyperpolarized and elongated mitochondrial networks and higher mitochondrial ROS concentration and increased oxidative phosphorylation, when exposed to a galactose (GAL)-containing cell culture medium [179]. Dynamin-related protein 1 (DRP1) is a critical factor in regulating mitochondrial dynamics. Notably, the oxidative stress-inducible kinase c-Abl phosphorylates DRP1 and augments the GTPase activity of DRP1 promoting DRP1-mediated mitochondrial fragmentation [180]. Thus, c-Abl/DRP1 signaling regulates oxidative stress-induced mitochondrial fragmentation and cell death [181]. In contrast, blocking DRP1 improves both mitochondrial function and autophagic flux in experimental PD models [181]. Furthermore, DRP1 inhibition reduces exosome release and spread of *α*-syn pathology from neurons to neurons and from microglia to neurons linking oxidative stress to exosome traffic [181].

### 4.1. Milk Exosomes: Potential Modifiers of Chaperone-Mediated Autophagy

Both mTORC1-regulated macroautophagy and chaperone-mediated autophagy (CMA) regulate cellular *α*-syn homeostasis [182,183,184,185,186,187]. Increasing evidence highlights the existence of a strong relationship between CMA defects and PD [188,189]. Wild-type *α*-syn is selectively translocated into lysosomes for degradation by the CMA pathway [183]. The monomeric form of *α*-syn is predominantly degraded by CMA, and Ser-129 phosphorylation of *α*-syn leads to its degradation via the proteasome system [186]. The lysosome-associated membrane protein type 2A (LAMP2A) assists with protein-lysosomal docking, internalization, and final degradation, and is involved in the clearance of damaged proteins including *α*-syn [187]. It has been demonstrated in the Drosophila brain that LAMP2A promotes autophagic flux and prevents *α*-syn-induced PD-like symptoms [190]. The selective loss of LAMP2A in the early stages of PD correlates with increased levels of *α*-syn [191]. The expression level of LAMP2A is significantly reduced in the SN pars compacta and amygdala of PD brains compared with age-matched controls [192]. Decreased LAMP2A levels in dopaminergic cell lines reduce CMA activity and increase the half-life of *α*-syn [192]. Decreased LAMP2 concentrations in CSF have also been found in female PD patients with leucine-rich repeat kinase 2 (*LRRK2*) mutations [193]. CMA also plays a role in direct degradation of neuronal transcription factor MADS box transcription enhancer factor 2, polypeptide D (MEF2D), a protein known to promote neuronal survival. Disruption of this regulatory pathway by *α*-syn leads to neuronal stress, which may underlie neuronal loss in PD [194].

Of importance, increased levels of miRNA-21, which directly targets the 3’UTR of LAMP2A mRNA [195], have been detected in SN of PD patients [196]. Thus, decreased CMA caused by miRNA-21-induced downregulation of LAMP2A may play an important role in disturbed *α*-syn homeostasis in PD [195,196]. Furthermore, the miRNA-21/LAMP2A axis is used for pharmacological intervention in PD as shown for geniposide that reduces *α*-syn by blocking miRNA-21/LAMP2A interaction in PD models [195]. In addition, downregulation of miRNA-21 protects cells from 1-methyl-4-phenylpyridinium (MPP)-mediated cytotoxicity by the inhibition of apoptosis induction, the reduction of the inflammatory response and the suppression of ROS production [197]. It has been demonstrated in SH-Y5Y cells that docosahexaenoic acid and aspirin exerted a synergetic neuroprotective effect by inhibiting miRNA-21 expression and activating retinoid X receptor *α* (RXR*α*) and peroxisome proliferator-activated receptor *α* (PPAR*α*) [198]. Modulation of LAMP2A expression and consecutive CMA activity is regarded as a therapeutic target for PD and other synucleinopathies [199]. Of note, increased exosomal miRNA-21 signaling is a common feature of malignant melanoma and glioblastoma multiforme [200,201], malignancies that exhibit higher rates of occurrence in patients with PD [202,203].

Postnatal exposure of intestinal cells including EECs with breastmilk exosome-derived miRNA-21 and resulting CMA suppression may be a timely restricted physiological mechanism to increase cellular *α*-syn levels for neuronal growth, synaptic and cognitive development during the breastfeeding period. However, persistent exposure of humans with bovine MEX and their miRNA-21 cargo may disturb LAMP2A-dependent CMA promoting exosome-mediated egress of misfolded *α*-syn via the vagal nerve to the SN. Furthermore, MEX and their miRNA-21 cargo may directly target SN LAMP2A via the circulatory route [133,204]. In fact, plasma concentrations of bovine miRNA-21 were >100% higher 6 h after consumption of 1.0 L 1%-fat commercial cow’s milk in healthy human volunteers [205].

Taken together, MEX miRNA-21/LAMP2A signaling in EECs may promote the vagal transfer of misfolded *α*-syn to the brain stem, a potential regulatory impact of milk on the gut–brain axis [26,27,28,29,30,31,32,33], whereas bovine MEX that reach the brain via systemic circulation [133] may additionally impair CMA homeostasis in the CNS.

AMPK-induced autophagy may be further attenuated by MEX miRNA-148a. It has been demonstrated that the upregulation of miRNA-148a inhibits the expression of AMPK [206]. In a highly conserved manner with strong binding affinity, miRNA-148a targets the catalytic subunit *α*1 of AMPK (*PRKAA1*) as well as the AMPK regulatory subunit γ2 (*PRKAG2*) (targetscan.org). MiRNA-148a-mediated downregulation of AMPK increases mTORC1 signaling thereby decreasing ULK1-mediated autophagy.

**Figure 2:**
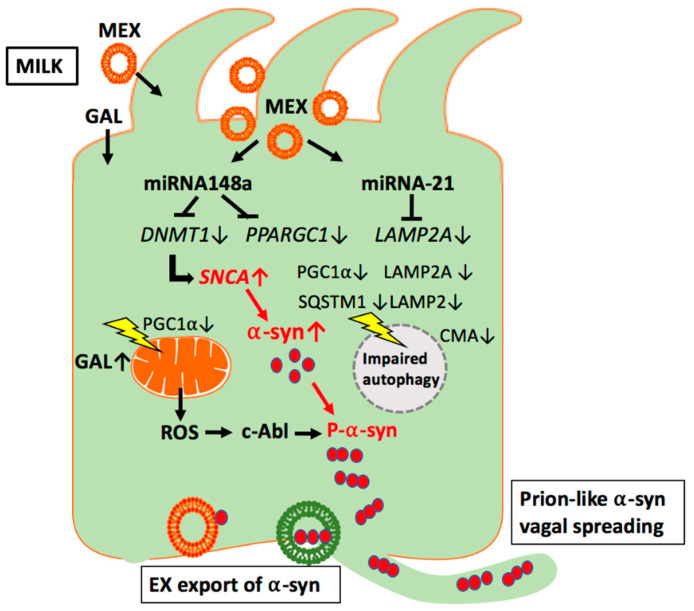
Working model of milk exosome (MEX) miRNA-mediated suppression of autophagy and mitochondrial function in enteroendocrine cells. MEX miRNA-148a may inhibit autophagy and mitochondrial function via suppression of peroxisome proliferator-activated receptor γ coactivator 1*α* (PGC1*α*). In addition, MEX miRNA-21 may inhibit chaperone-mediated autophagy (CMA) via suppression of lysosome-associated membrane protein 2A (LAMP2A). Increased release of reactive oxygen species (ROS) via miRNA-148a- and galactose (GAL)-mediated impairment of mitochondrial function may activate the ROS-sensitive kinase c-Abl, which phosphorylates *α*-syn promoting its aggregation. Excessive *α*-syn oligomers are preferentially excreted by exosomal release and spread either via the vagal nerve route or systemic circulation.

Under conditions of deficient lysosomal autophagy and oxidative stress, only partial degradation of monomeric and fibrillar forms of *α*-syn occurs. Accumulation of C-truncated monomeric *α*-syn may kick start initial aggregation and fibril formation, leading to the prion-like seeding cycle of pathology propagation [207]. It is conceivable that due to diminished lysosomal activity induced by MEX signaling, accumulated C-truncated and aggregated *α*-syn is exported via exosome release contributing to the prion-like spread of toxic *α*-syn [207,208] (Figure 2).

### 4.2. Bacterial Fermentation Degrades Milk Exosomes

Fermentation of raw cow’s milk with various strains of lactic acid bacteria reduces exosome size, protein content and results in a substantial loss of miRNAs including miRNA-21 [209]. Notably, MEX after fermentation compared to untreated MEX exhibit reduced proliferation when added to IEC-6 cells [209]. The impact of bacterial fermentation on MEX and their miRNA content may explain the epidemiological differences between non-fermented and fermented milk observed in patients with PD (Table 1).

## 5. Milk and Oxidative Stress

### 5.1. Galactose: Inducer of Oxidative Stress

Human breastmilk provides only small amounts of free galactose (GAL) (0.013 g/100 mL), but high amounts of lactose (galactose-1,4-glucose) (7.26–7.92 g/100 mL) that do not change during the first 6 months of breastfeeding [210]. Liver glycogen synthesis in infants is formed mainly from breastmilk-derived GAL [211] and acts as an energy reservoir for subsequent hepatic glucose release to the circulation during times of fasting [212,213]. In all mammals, milk-derived GAL supply abruptly declines after the breastfeeding period except in humans who continuously consume cow’s milk and dairy products. The liver plays the central role in the first-order clearance of plasma GAL [214]. Physiologically, the GAL elimination capacity (GEC) is significantly higher in healthy children than in healthy adults, diminishing to adult levels by the age of 16 years [215]. In adults, GEC is related to body weight and decreases slowly with age [216,217]. Among patients with newly-diagnosed cirrhosis and decreased GEC, GEC is a strong predictor of short- and long-term all-cause and cirrhosis-related mortality [218]. There is accumulating evidence that GAL increases oxidative stress, which has recently been linked to increased all-cause mortality by consumption of non-fermented milk [219,220].

### 5.2. Galactose: A Mitochondrial Stressor

Mitochondrial dysfunction and oxidative stress are hallmarks in the pathogenesis of PD [13,221,222,223]. In a normal functioning neuron, mitochondria supply cellular adenosine triphosphate (ATP) via the respiratory chain [224]. Dopaminergic neurons produce and consume a great amount of ATP to maintain synapse connections pointing to an important role of mitochondria for neuronal health [225,226,227]. Mitochondria are undoubtedly changed in PD, and mitochondrial functions are disrupted in genetic and pharmacologic models of PD [179,225,228]. Remember, MPTP, which is a well-known mitochondrial toxin, induces PD-like motor symptoms [229,230,231].

GAL is another mitochondrial stressor experimentally used for the induction of brain aging and neurodegeneration [232,233]. Several studies have shown that GAL induces brain aging, mitochondrial dysfunction, oxidative stress, inflammation, apoptosis, as well as lowering brain-derived neurotrophic factors [234]. Recent evidence indicates that ginsenoside Rg1 decreases oxidative stress and downregulates Akt/mTORC1 signaling to attenuate cognitive impairment in mice and senescence of neural stem cells induced by GAL [235]. Brain aging is significantly associated with mitochondrial dysfunction characterized by a decrease in the activity of respiratory chain enzymes and ATP production, increased free radical generation, mitochondrial DNA mutations, and impaired mitochondrial structures [234,236].

González-Casacuberta et al. [237] recently analyzed mitochondrial function and autophagy in skin fibroblasts of *PRKN* mutation-associated PD in standard (glucose) and mitochondrial-challenging (GAL) conditions. In glucose, *PRKN*-PD fibroblasts show preserved mitochondrial bioenergetics with trends to abnormally enhanced mitochondrial respiration that, accompanied by decreased complex I, may account for the increased oxidative stress. In GAL, *PRKN*-PD fibroblasts exhibited decreased basal/maximal respiration vs. controls and reduced mitochondrial complex IV and increased oxidative stress compared to glucose, suggesting an inefficient mitochondrial oxidative capacity to meet an extra metabolic requirement [237]. In addition, exhaustion of mitochondrial bioenergetic and autophagic reserve has been associated with the development of PD in *LRRK2*^G2019S^ mutation carriers [238].

### 5.3. Galactose: Inducer of miRNA-21

In a GAL-induced pseudo-aging mouse model, a significant increase of miRNA-21 has been observed, whereas miRNA-21 knockout mice were resistant to GAL-induced alterations in aging-markers and cardiac function [239]. Treatment of rat spinal cord neurons with hydrogen peroxide, a GAL-induced ROS, upregulates the expression of miRNA-21 [240]. In analogy, recent evidence supports the association of increased cellular miRNA-21 expression with oscillating and high glucose exposure disrupting mitochondrial homeostasis [241]. GAL-induced expression of miRNA-21 may suppress LAMP2A-dependent CMA, an unfavorable constellation further increasing intracellular levels of *α*-syn. Moreover, GAL-induced mitochondrial ROS may enhance *α*-syn misfolding and aggregation [242,243]. In fact, increased ROS generation triggers mitochondrial importation of *α*-syn subsequently inducing intra-mitochondrial *α*-syn aggregation and respiratory complex I dysfunction. In a vicious cycle, overexpressed or mutated *α*-syn will further impair mitochondrial function with subsequent ROS production promoting conformational changes of *α*-syn [244,245].

### 5.4. Oxidative Stress and Abelson Tyrosine Kinase Activation

Kinases of the c-Abl family play a role in the development of the CNS. c-Abl (ABL1; Abelson tyrosine kinase) is a member of Abl family of non-receptor tyrosine kinases and is in the scope of recent research in PD [246]. Increased c-Abl activation, primarily induced by oxidative stress, is reported in PD [247,248,249]. Activated c-Abl emerges as a common link to various PD-related inducers of oxidative stress relevant to both sporadic and familial forms of PD and *α*-synucleinopathies [246,247,248]. c-Abl regulates the degradation of parkin and *α*-syn, both involved in PD pathogenesis. The inhibition of parkin’s neuroprotective functions is regulated by c-Abl-mediated phosphorylation of parkin [250]. c-Abl directly catalyzes *α*-syn phosphorylation mainly at tyrosine-39 (Tyr39) [250]. Analysis of human brain tissues showed that Tyr39 *α*-syn is detected in the brains of healthy individuals and those with PD [250]. However, only c-Abl protein levels were found to be upregulated in PD brains [250]. Importantly, phosphorylation of *α*-syn at Tyr39 directly impairs the interaction of *α*-syn with chaperones, thus providing a functional explanation for the role of c-Abl in PD [251]. A short motif around Tyr39 was identified as being crucial for the aggregation of *α*-syn. Interestingly, this region is also one of the main segments in contact with a diverse pool of molecular chaperones. Furthermore, inhibition of the chaperone/*α*-syn interaction leads to binding of *α*-syn to mitochondria resulting in mitochondrial membrane disruption [252]. Interestingly, nilotinib, a specific inhibitor of c-Abl kinase activity, induces *α*-syn protein degradation via the autophagy and proteasome pathways, whereas the overexpression of *α*-syn in the rat midbrains enhances c-Abl expression [252].

Thus, milk GAL-induced oxidative stress may increase c-Abl-mediated *α*-syn phosphorylation reducing *α*-syn/chaperone interaction, whereas GAL-induced expression of miRNA-21 reduces LAMP2A-mediated CMA, two adverse synergetic events that impair lysosomal clearance of *α*-syn, a constellation boosting exosome-mediated *α*-syn export.

### 5.5. Bacterial Fermentation Reduces Galactose Content of Dairy Products

The total GAL content of bovine milk is 2.4 g/100 g [253]. The total GAL content of yogurt is 94–95% of that in milk, whereas, in Swedish soured milk and kefir, GAL content is in the range of 75 to 79% of that in milk [253]. The GAL content of cheese depends on the fermentation procedure. The recommended GAL content of cheese for patients with galactosemia should not exceed 10 mg/100 g [254,255]. Whereas most strains of *S. thermophilus* cannot metabolize the GAL moiety of lactose [256], lactococci are able to degrade glucose and GAL by the glycolytic and Leloir pathway [257,258]. All of the lactic streptococci examined except *Streptococcus lactis* ML8 ferment GAL to lactate, formate, acetate, and ethanol [259]. It has recently been demonstrated in 1053 individuals with idiopathic PD that both yogurt and cheese consumption were associated with a more rapid progression of PD [260]. Thus, milk and fermented milk products are significant dietary sources of GAL, critical inducers of ROS, ROS-dependent Abl-c activation, miRNA-21 expression, and mitochondrial dysfunction that adversely affect lysosomal clearance of altered and excessive *α*-syn.

### 5.6. Branched-Chain Amino Acids and Oxidative Stress

Compared to other plant and animal protein sources, milk proteins contain the highest amounts of branched-chain amino acids (BCAAs) leucine, isoleucine and valine [261], key drivers for mTORC1 activation [74,75,76]. Recent evidence indicates that deficits in BCAA catabolism may increase mitochondrial activity levels early in PD neurons. The resulting oxidative damage may drive the ultimate loss of mitochondrial function, eventually leading to neuronal cell death [262]. Remarkably, branched-chain amino transferase 1 (BCAT1) has been identified as a new player in PD pathogenesis [263]. BCAT1 expression is significantly decreased in the SN of sporadic PD patients, and RNAi-mediated knockdown of bcat-1 in *C. elegans* causes an age-dependent, progressive motor disorder and promotes dopaminergic neurodegeneration in worms expressing human *α*-syn [263]. Of note, BCAT1 expression decreases during normal aging in worms, fish, and mice [264]. BCAT1 knockdown increases mitochondrial respiration and induces oxidative damage in neurons through mTOR-independent mechanisms. Mitochondrial hyperactivity is related to BCAT1(RNAi) neurotoxicity, whereas metformin reduces mitochondrial respiration to control levels and significantly improves both motor function and neuronal viability [93,265].

Particularly in the context of the age-dependent decline of BCAT1 activity, increased dairy protein intake enriched in BCCAs may further impair mitochondrial function, which is of critical importance for dopaminergic neurons and *α*-syn homeostasis. Remarkably, cumulative intake of BCAAs and diminished BCAA catabolism plays also a key role in the pathogenesis of T2D [266,267,268,269], whereas a restriction of BCAAs improves metabolic health and glucose homeostasis [270,271,272,273].

### 5.7. MiRNA-148a: Inhibitor of Mitochondrial Function and Autophagy

MiRNA-148a is an abundant miRNA species (signature RNA) detected in cow’s milk [118], milk lipids [274], bovine EV fractions [116,117,275], and human and bovine MEX [114]. *MIR148A* is a domestication gene of dairy cows enhancing lactation performance and milk yield [276]. As shown in goat mammary epithelial cells, miRNA-148a increases milk triacylglycerol synthesis via targeting *PPARGC1A* (peroxisome proliferator-activated receptor γ coactivator 1*α* (PGC-1*α*) [277].

PGC-1*α* is a key transcriptional regulator in tissues that undergo extensive oxidative metabolism and operates as a central organizer of metabolic function, oxidative states, and mitochondrial biogenesis and function [278]. Intriguingly, it has been observed in the MPTP mouse model of PD, which is associated with increased oxidative stress and mitochondrial dysfunction [279], that MPTP significantly increases the expression of miRNA-148a [173].

In contrast, activation or stabilization of PGC-1*α* maintains mitochondrial functions and renders mice resistant to MPTP-induced PD [280,281,282,283]. Remarkably, a reduction in PGC-1*α* reduces mitochondrial membrane potential, intracellular ATP content and intracellular H_2_O_2_ generation, leading to the translocation of cytochrome c to the cytoplasm in the MPP-induced PD cell model [284]. PGC-1*α* has a significant impact on mitochondrial signal transduction in dopaminergic neurons by upregulating the expression of estrogen-related receptor *α* (ERR*α*), NRF-1, NRF-2 and PPARγ [283]. Both ERR*α* and PGC-1*α* cooperate to induce mitochondrial biogenesis [285]. For instance, PGC-1*α* interacts with ERR-*α* and recruits it to the ERR-*α* response element motif located in the proximal *MPC1* (mitochondrial pyruvate carrier 1) promoter activating *MPC1* expression, which is essential for mitochondrial pyruvate usage and mitochondrial ATP production [286]. In accordance, the addition of an EER agonist to culture media enhances glycolysis and mitochondrial respiration leading to elevated cellular ATP levels [287].

Recent evidence underlines that PGC-1*α* plays also a central role in the regulation of autophagy [288]. As demonstrated in vascular smooth muscle cells (VSMCs) of mice PGC-1*α*-deficiency results in abnormal and reduced numbers of autophagosomes associated with reduced expression of LC3-II (microtubule-associated protein 1A/1B-light chain 3-II), SQSTM1 (sequestosome 1 = ubiquitin-binding protein p62), LAMP2 (lysosome-associated membrane protein 2), CTSD (cathepsin D), and TFRC (transferrin receptor) [288]. The selective autophagy receptor p62/SQSTM1 is important for the regulation of the selection of proteins or organelles for degradation [289,290]. Dysregulation of SQSTM1/p62 has been related to the development of a variety of neurodegenerative disorders including PD [291,292]. Notably, p62/SQSTM1 is an interacting partner of LRRK2 [293]. The pathogenic *LRRK2* mutations (N1437H, R1441C/G/H, Y1699C, G2019S) associated with familial forms of PD increase phosphorylation of p62 and thereby enhance neurotoxicity [293] (Figure 2).

Persistent signaling of milk-derived miRNA-148a via targeting PGC-1*α* may thus increase oxidative stress and attenuate autophagy of misfolded proteins. These epigenetic milk-derived alterations may adversely affect *α*-syn homeostasis in EECs but also in distant regions such as highly energy-dependent dopaminergic neurons of the SN and pancreatic *β*-cells, which as well are exposed to systemic exosome traffic [294,295,296,297,298,299] and vagal connectivity [300,301] (Table 2).

## 6. The *α*-Synuclein Link between Parkinson’s Disease and Type 2 Diabetes

Epidemiological evidence suggests a link between PD and T2D [35,36,37,38,39,302]. T2D is a negative prognostic factor associated with faster motor progression and cognitive decline in PD. The presence of T2D in individuals without PD is associated with PD-like pathology including striatal dopaminergic deficits and increased CSF levels of tau and *α*-syn [303]. Nearly 60% of patients with PD are insulin resistant. Recently, Martinez-Valbuena et al. [304] found cytoplasmic phosphorylated *α*-syn deposits in the pancreatic *β*-cells in 93% of PD patients, in 85% of subjects with LB dementia and in 73% of incidental LB disease. Similar phosphorylated *α*-syn inclusions were found in 68% of subjects with a normal neuropathological examination but with T2D compared to 17% in healthy controls [304]. 

### 6.1. α-Synuclein: Functional Component of β-Cells

In the *β*-cell, *α*-syn interacts with K-ATP channels and insulin-secretory granules and functionally acts as a brake on secretion that glucose stimulation can override [305]. *α*-Syn normally acts to inhibit insulin secretion from pancreatic *β*-cells by interacting with the Kir6.2 subunit of the ATP-sensitive potassium channel (K-ATP). It is also known that K-ATP channels act to inhibit brain dopamine secretion, and *α*-syn is a normal inhibitor of dopamine synthesis [306].

*β*-cells exist in the context of a complex, integrated pancreatic islet microenvironment where they interact with other endocrine cells, vascular endothelial cells, extracellular matrix, islet macrophages and neuronal projections [307]. *β*-cell proliferation is stimulated by parasympathetic and inhibited by sympathetic signals [300,301,308]. Neuronal signals regulate obesity-induced *β*-cell proliferation by a forkhead box M1 (FoxM1) dependent mechanism [309]. Enteric neurons of the gut project to the pancreas and moreover the pancreas is densely innervated by the vagal nerve leaving open the possibility of *α*-syn transmission to the pancreas via the enteric nervous system and/or the vagal nerve [310]. In fact, vagotomy ameliorates islet morphofunction and body metabolic homeostasis in monosodium glutamate-obese rats [311].

T2D, like PD, belongs to the group of protein misfolding diseases (PMDs), which share aggregation of misfolded proteins as a hallmark. Although the major aggregating peptide in *β*-cells of T2D patients is islet amyloid polypeptide (IAPP), *α*-syn in *β*-cells cross-reacts with IAPP in vitro [312]. Recently, Mucibabic et al. [310] showed that *α*-syn is a component of amyloid extracted from the pancreas of transgenic mice overexpressing human IAPP (denoted hIAPPtg mice) and from islets of T2D individuals. Notably, *α*-syn dose-dependently promoted IAPP fibril formation in vitro and after tail-vein injection of *α*-syn in hIAPPtg mice enhanced *β*-cell amyloid formation in vivo, whereas *β*-cell amyloid formation was reduced in hIAPPtg mice on a *SNCA*-/- background [310]. It is not yet known whether Tyr39-phosphorylation of *α*-syn is responsible for the co-aggregation of *α*-syn with IAPP. It is well appreciated that aggregated IAPP has cytotoxic properties and is believed to be of critical importance for the loss of *β*-cells in T2D [313]. Excessive production and aggregation of IAPP eventually promoted by *α*-syn increases endoplasmic reticulum stress and disrupts autophagy, critical events involved in *β*-cell death in T2D [314,315].

### 6.2. Galactose Disturbs β-Cell α-Synuclein Homeostasis

Glucose transporter 2 (GLUT2) is important for the handling of dietary sugars including glucose, fructose and GAL [316]. GLUT2 is expressed in enterocytes, pancreatic islet *β*-cells as well as in CNS [317,318]. GAL is a reducing sugar that can be metabolized at a normal concentration. However, at high levels, GAL can be converted into aldose and hydroperoxide under the catalysis of GAL oxidase, resulting in the generation of a superoxide anion and oxygen-derived free radicals [319,320,321,322,323,324]. In mice receiving GAL (500 mg/kg) daily by oral gavage for 6 weeks, glucose, insulin, insulin resistance significantly increase along with a significant decrease in superoxide dismutase activity and pancreatic islet insulin secretion [325]. Thus, persistent excessive intake of milk combined with other dairy-derived sources of GAL may increase oxidative stress in pancreatic *β*-cells. In analogy with neuronal cell oxidative stress in PD, oxidative stress of *β*-cells plays a key role in the pathogenesis of T2D [326].

Of crucial importance in the pathogenesis of T2D is autophagy, in particular the removal of dysfunctional mitochondria via mitophagy, a form of macroautophagy selective for mitochondria [327,328,329]. Cultured HepG2 cells grown in glucose media produce their ATP by glycolysis, largely bypassing the mitochondria, and hence are fairly resistant to drugs that affect mitochondrial function. However, when growing the same cells in media supplemented with GAL as opposed to glucose, they are forced to produce ATP through oxidative phosphorylation, which then makes them vulnerable to mitochondrial insults [330]. Remember, GAL-induced mitochondrial dysfunction and resulting oxidative stress has been implicated to play an important role in the activation of c-Abl [236].

Intriguingly, imatinib mesylate, a selective tyrosine kinase inhibitor, targets c-Abl, antagonizes ABL–IRE1*α* interaction, blunts IRE1*α* RNase hyperactivity, reduces pancreatic *β*-cell apoptosis, and reverses type 1 diabetes (T1D) in the non-obese diabetic (NOD) mouse model [331]. Imatinib also induces regression of T2D [332,333]. Recent evidence indicates that imatinib enhances insulin production by *β*-cells [334]. Notably, c-Abl negatively regulates insulin production via interfering with the expression of NKx2.2 and GLUT-2 [334]. Remarkably, imatinib is known to possess anti-amyloid properties in AD models [335]. The putative anti-amyloid/fibrotic effect of imatinib in T2D has been linked to its inhibitory effect on c-Abl [336].

**Figure 3:**
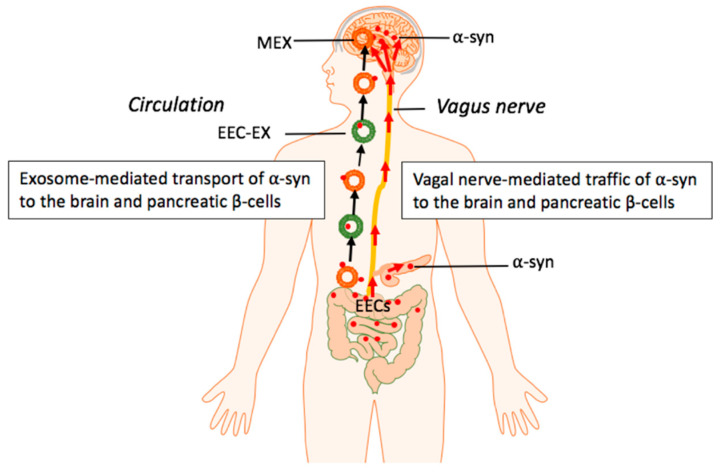
Hypothetical model: Milk exosome (MEX) miRNAs promote intestinal synthesis and impaired catabolism of *α*-syn with transmission of aggregated *α*-syn via the vagal nerve and systemic circulation of enteroendocrine cell (EEC)-derived exosomes as well as MEX that may reach both pancreatic *β*-cells and dopaminergic neurons of the brain.

*IDE* is a T2D risk gene located at the *HHEX/IDE* T2D locus. *IDE* encodes an intracellular metalloprotease called insulin-degrading enzyme (IDE), which is responsible for the elimination of proteins with amyloidogenic potential [337]. Notably, decreased IDE levels are associated with increased levels of *α*-syn in human T2D islets [338]. Increased expression of *α*-syn in *β*-cells of normal mice impairs glucose-stimulated insulin secretion (GSIS) and autophagic flux underlining the diabetogenic effect of increased *β*-cell *α*-syn expression. It has been demonstrated that IDE operates like a chaperone of *α*-syn and prevents the formation of *α*-syn amyloid fibrils [339]. Excessive endogenous synthesis of *α*-syn in the pancreatic *β*-cell and/or transfer of toxic *α*-syn from the gut the *β*-cell via the vagal route [300,301,309] or transfer via *α*-syn-enriched plasma exosomes in PD patients [340] are potential pathways that may promote T2D in patients with PD (Figure 3).

## 7. Conclusions and Perspectives

The epidemiological link between milk consumption and PD and T2D points to a common underlying pathogenic mechanism. Both chronic and frequently associated diseases belong to the family of misfolded protein diseases sharing aggregated *α*-syn as a common denominator. The important physiological function of *α*-syn for postnatal neural development and milk’s evolutionary mission for postnatal growth and neuronal differentiation have been overlooked in the past. MEX transmitting milk’s signature miRNAs, miRNA-148a and miRNA-21, may epigenetically increase intracellular *α*-syn levels either by enhanced *α*-syn expression and/or decreased *α*-syn degradation. Persistent intake of milk- and dairy-derived GAL, a well-known inducer of oxidative stress, especially with advanced age associated with decreased hepatic GAL elimination, may promote c-Abl-mediated *α*-syn aggregation. The intrinsic signaling capacity of pasteurized milk, but less likely a toxic pollutant in milk, apparently functions as the critical environmental mechanism promoting PD and T2D [205]. In accordance with CSF exosomes of PD patients [341], it is tempting to speculate that MEX may assist in *α*-syn transmission and may aggravate *α*-syn aggregation and consecutive exosomal spreading to adjacent cells. The inhibition of autophagy by milk-mediated activation of mTORC1 and the impairment of autophagy and CMA via MEX miRNA-148a/miRNA-21 signaling may stimulate exosomal egress of *α*-syn to the brain and eventually to the pancreatic islets. The detection of MEX in the brain of mice after oral administration of bovine MEX allows the prediction that MEX have a direct impact on the gut–brain axis. Either via the vagal nerve and/or the blood circulation, exosomal *α*-syn may reach dopaminergic cells of SN as well as pancreatic *β*-cells. The physiological function of milk and MEX during the breastfeeding period may thus turn into a hostile constellation promoting both *α*-synucleopathies, when milk signaling is not discontinued as originally programmed by mammalian physiology.

With the introduction of pasteurization of milk combined with the widespread availability of refrigeration technology, MEX unnoticeably entered the human food chain, modify the dietary exposome and disturb epigenetic regulation of the milk consumer. Thus, exosomes significantly contribute to the pathogenesis, progression and therapy of neurodegenerative diseases [150,342,343] and T2D [344,345,346]. Future research investigating MEX early impact on *α*-syn homeostasis during the physiological breastfeeding period and their long-term abuse in the pathogenesis of neurodegenerative diseases and T2D might offer new perspectives for the prevention and treatment of these common and related diseases of Western civilization (Table 3).

## 8. Materials and Methods

A non-systematic approach was chosen and a narrative synthesis of the results of the searched articles was carried out in accordance with Gasparian et al. [347] and Saracci et al. [348].

## Figures and Tables

**Table 1:** Epidemiological evidence for milk/dairy intake and risk of Parkinson’s disease.

Total Dairy	Milk	Cheese	Yogurt	References
**+**	**+**			Chen et al. (2002) [40]
	**+**			Park et al. (2005) [41]
	**+**			Sääksjärvi et al. (2013) [42]
	**+**			Kyrozis et al. (2013) [43]
	**+**	**+**		Jiang et al. (2014) [44]
	**+**			Hughes et al. (2017) [45]
	**+**			Olsson et al. (2020) [46]

**Table 2:** The potential impact of milk-derived exosomes (MEX) on *α*-synuclein pathology.

Agent	Predicted Functions	References
MEX miRNA-148a	Suppression of DNMT1, hypomethylation of *SNCA* with increased expression of *α*-syn in EECs and SN neuron.	[114,125]
	Suppression of AMPK activity increasing mTORC1 activity resulting in reduced ULK-1-mediated autophagy.	[206]
	Suppression of PGC-1α reducing the expression of LC3-II, SQSTM1 and LAMP2 resulting in reduced autophagy.	[288]
	Suppression of PGC-1α-mediated mitochondrial function with increased oxidative stress promoting *α*-syn aggregation.	[280,281,282,283,284,285]
MEX miRNA-21	Reduction of DNMT1 activity, hypomethylation of *SNCA* with increased expression of *α*-syn in EECs and SN neurons.	[126]
	Suppression of LAMP2A resulting in reduced chaperone-mediated autophagy, accumulation of *α*-syn promoting *α*-syn aggregation.	[195,196,197]
Exosome lipids	Promotion of *α*-syn aggregation.	[149,158]
Exosome membrane	*α*-syn binding to exosome outer membrane promoting exosome *α*-syn spreading.	[152]

**Table 3:** Overlapping pathogenic factors in Parkinson’s disease (PD) and type 2 diabetes mellitus (T2D).

Pathogenic Factors	PD	References	T2D	References
Exosome traffic	**+**	[149,150,151,156,158,227,340,341,342,343]	**+**	[294,295,296,297,298,299,344,345,346]
Vagal connectivity	**+**	[20,21,22,23,24,27,28,29]	**+**	[300,301,308,311]
*α*-Synuclein aggregation ↑	**+**	[134,145,191]	**+**	[311]
Mitochondrial function ↓	**+**	[13,179,184,221,225,228,262]	**+**	[328]
Oxidative stress ↑	**+**	[222,223,237,238,242]	**+**	[326]
Autophagy ↓	**+**	[94,154,177,178,183,185,188,189,190,191,192,196]	**+**	[327,328,329]
Increased mTORC1 ↑	**+**	[82,83,84,85]	**+**	[57,58,266]
AMPK ↓	**+**	[90]	**+**	[59,60]
MiRNA-148a ↑	**+**	[173]	**+**	[206]
MiRNA-21 ↑	**+**	[196]	**+**	[241,266]
BCAA ↑	**+**	[263,265]	**+**	[268,269,270,271,272,273]
Metformin response	**+**	[89,90,91,92,93,265]	**+**	[61,62]

## Data Availability

The data presented in this study are openly available and are provided in the presented PubMed-based reference list.

## Abbreviations

ABL1: ABL protooncogene 1, nonreceptor tyrosine kinase; AD, Alzheimer’s disease; ALP, autophagy-lysosome pathway; AMPK, adenosine monophosphate-activated protein kinase; ATP, adenosine triphosphate; BBB, blood-brain barrier; BCAA, branched-chain amino acid; BCAT1, branched-chain amino transferase 1; CMA, chaperone-mediated autophagy; CNS, central nervous system; CSF, cerebrospinal fluid; DNMT1, DNA methyltransferase 1; DRP1, dynamin-related protein 1; EEC, enteroendocrine cell; EPIC, European Prospective Investigation into Cancer and Nutrition; ERR*α*, estrogen-related receptor *α*; EV, extracellular vesicle; FoxM1, forkhead box M1; FTO, fat mass- and obesity-associated gene; GAL, galactose; GEC, galactose elimination capacity; GI tract, gastrointestinal tract; GLUT2, glucose transporter 2; GSIS, glucose-stimulated insulin secretion; IDE, insulin-degrading enzyme; IGF-1, insulin-like growth factor 1; IAPP; islet amyloid polypeptide; IEC, intestinal epithelial cell; LAMP2A, lysosomal-associated membrane protein 2, isoform A; LB, Lewy bodies; LRRK2, leucine-rich repeat kinase 2; MEF2D, MADS box transcription enhancer factor 2, polypeptide D; MEX, milk exosome; miRNA, micro-ribonucleic acid; MPP, 1-methyl-4-phenylpyridinium; MPTP, 1-methyl-4-phenyl-1,2,3,6-tetrahydropyridine; mTORC1, mechanistic target of rapamycin complex 1; NOD, non-obese diabetic; NRF2, nuclear factor erythroid 2-related factor 2; PD, Parkinson disease; PMD, protein misfolding disease; PINK1, PTEN-induced putative kinase 1; PGC-1*α*, peroxisome proliferator-activated receptor γ coactivator 1*α*; PPAR*α*, peroxisome proliferator-activated receptor *α*; PPARγ, peroxisome proliferator-activated receptor γ; PRKN, Parkin; PTEN, phosphatase and tensin homolog; RAB11A, RAS-associated protein RAB11A; ROS, reactive oxygen species; RXR*α*, retinoid X receptor-*α*; SN, substantia nigra; SNARE, soluble N-ethylmaleimide-sensitive factor attachment protein receptor; SNCA, *α*-synuclein gene; *α*-Syn, *α*-synuclein; T1D, type 1 diabetes mellitus; T2D, type 2 diabetes mellitus; UTR, untranslated region.
